# Prediction Models for Delayed Bleeding After Colorectal Endoscopic Submucosal Dissection: A Systematic Review and Critical Appraisal

**DOI:** 10.1002/deo2.70456

**Published:** 2026-10-19

**Authors:** Da Miao, Zhuoer Hou, Yao Ge, Haiou Qi

**Affiliations:** ^1^ Nursing Department, Sir Run Run Shaw Hospital Zhejiang University School of Medicine Hangzhou China

**Keywords:** colorectal endoscopic submucosal dissection, critical appraisal, delayed bleeding, prediction models, systematic review

## Abstract

**Background:**

Although many predictive models have been developed or validated for delayed bleeding after colorectal endoscopic submucosal dissection (ESD), their methodological quality and clinical utility remain uncertain. We systematically reviewed and critically appraised all available predictive models for this outcome.

**Methods:**

We searched five databases through March 2026 for observational studies that developed or validated prediction models or scoring systems for delayed bleeding after colorectal ESD in adults (≥18 years). Two reviewers independently extracted data and assessed risk of bias using PROBAST.

**Results:**

Of 25,177 records screened, five studies reporting five predictive models were included. The incidence of delayed bleeding ranged from 2.9% to 9.9%. The most commonly used predictors were lesion location and lesion size. Discrimination in derivation cohorts ranged from 0.726 to 0.890, internal validation areas under the curve [AUCs] ranged from 0.739 to 0.870, and the externally validated model achieved an AUC of 0.634. All studies were judged to have a high risk of bias, mainly because of inadequate reporting in the predictor, outcome, and analysis domains, although most had low concerns regarding applicability.

**Conclusions:**

This systematic review identified five predictive models for delayed bleeding after colorectal ESD. Lesion location and size were the most frequently reported predictors. Discriminative performance varied across derivation and validation cohorts, with suboptimal external‐validation results. All models had a high risk of bias per PROBAST (low concerns regarding applicability); therefore, their findings should be interpreted cautiously.

## 1 Background

Endoscopic submucosal dissection (ESD) has become a standard minimally invasive technique for the treatment of early colorectal cancer and precancerous lesions. Its advantage lies in enabling en bloc resection of lesions, with reported en bloc resection rates ranging from 89.2% to 92.5% [1, 2, 3]. Compared with conventional surgery, ESD offers less trauma, faster recovery, and shorter hospital stays [4]. However, the technical complexity of colorectal ESD—characterized by high difficulty, prolonged operation time, and submucosal dissection—increases the risk of vascular and muscular injury [5]. Consequently, complications are not entirely avoidable. Among these, delayed bleeding is one of the most common complications, clinically manifesting as melena or hematochezia after the procedure and, in severe cases, can lead to hemorrhagic shock. Its reported incidence ranges from 2.80% to 5.37% [6, 7], prolonging hospitalization, increasing costs, and threatening patient safety. Despite precise intraoperative hemostasis and prophylactic interventions, the lack of effective risk assessment tools prevents targeted individualized management for high‐risk patients, contributing to the recurrent occurrence of delayed bleeding [8].

Risk assessment tools for delayed bleeding after colorectal ESD can be categorized by the timing of predictor availability. Preoperative models, using variables available before the procedure, can inform patient selection and consent. In contrast, models incorporating intraoperative predictors, such as intraoperative bleeding or submucosal fibrosis, become available only during or after the procedure and are more suited for guiding post‐ESD surveillance or early intervention. Several predictive models have recently been developed, with some showing favorable discriminative ability (area under the curve [AUC] > 0.8) [9, 10, 11]. However, clinical adoption remains limited, potentially due to methodological limitations, predictor selection, model performance, and validation rigor. For instance, some studies have inadequate sample sizes, increasing the risk of overfitting [11]; others rely solely on univariate analysis for predictor screening, potentially omitting critical information from multivariate interactions [9, 10, 11, 12, 13]. Furthermore, most existing models lack rigorous external validation to substantiate their generalizability [9, 10, 11, 13]. Currently, there is a dearth of comprehensive synthesis, comparison, and evidence integration of these predictive models.

Therefore, this systematic review aims to systematically retrieve, evaluate, and synthesize the existing research evidence on predictive models for delayed bleeding after colorectal ESD. By comprehensively assessing their methodological design, predictive performance, strengths, and limitations, and clarifying their applicable scenarios based on the timing of predictor availability, this review provides evidence‐based references for clinicians to select optimal predictive tools, while also identifying directions for further optimizing models and developing standardized, clinically feasible tools.

## 2 Method

### 2.1 Study Design

This systematic review was prospectively registered in the International Prospective Register of Systematic Reviews prior to the commencement of literature searches (Registration ID: CRD420251252241).

### 2.2 Data Sources and Eligibility Criteria

Two independent researchers conducted a comprehensive search of five English and Chinese electronic databases from their inception to March 2026: PubMed, Web of Science, Embase, the Cochrane Library, and China National Knowledge Infrastructure. The following keywords were used to conduct a basic search: “delayed bleeding,” “bleeding,” “postoperative bleeding,” “delayed hemorrhage,” “postoperative hemorrhage,” “prediction model,” “prediction,” “predict model,” “risk prediction,” “risk factors,” “risk assessment,” “prognostic model,” “model,” “nomogram,” “endoscopy,” “endoscopic,” “endoscopic resection,” “endoscopic submucosal dissection,” “ESD,” “endoscopic mucosal resection,” “EMR.” Specific details regarding the strategies are in the Supporting Information. References from included studies and previous relevant systematic reviews were also checked to identify any additional eligible research. Figure 1 shows the process of screening articles.

**FIGURE 1:**
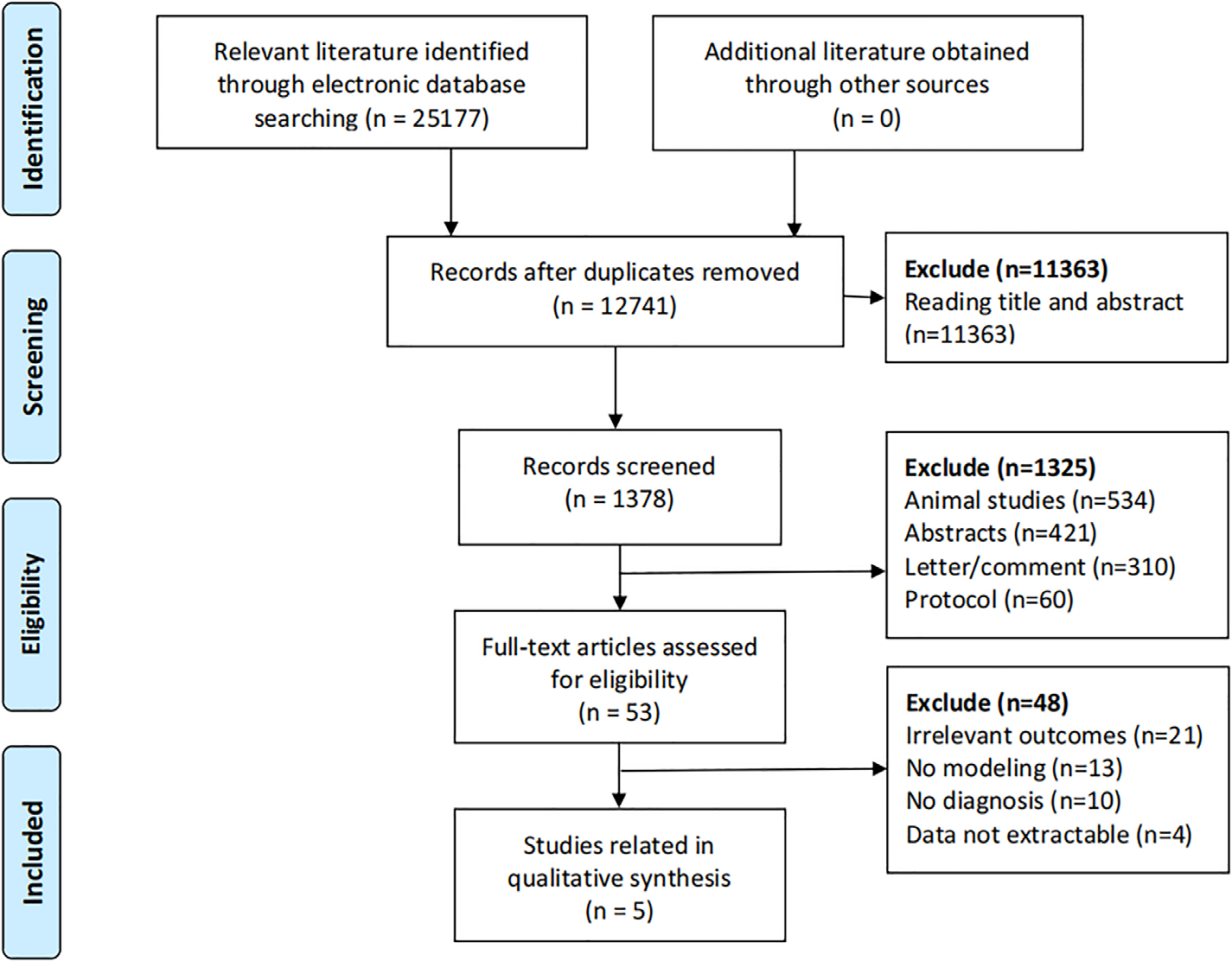
Flow diagram of literature selection process and results according to the Preferred Reporting Items for Systematic Reviews and Meta‐Analyses (PRISMA).

Eligibility criteria included observational studies that either developed or validated prediction models or scoring systems for predicting delayed bleeding in patients undergoing colorectal ESD. In accordance with the recommendations outlined in the Critical Appraisal and Data Extraction for Systematic Reviews of Prediction Modeling Studies (CHARMS) checklist [14], the PICOTS system was utilized to characterize the key components of our systematic review, which are presented as follows.

P (Population): The population of interest comprises patients undergoing colorectal ESD who are aged over 18 years old.

I (Intervention model): Studies focus on prediction models that have been internally or externally validated.

C (Comparator): None.

O (Outcome): Delayed bleeding after colorectal ESD. Delayed bleeding after colorectal ESD is defined as a clinical condition characterized by a decrease in hemoglobin level of at least 2 g/dL compared with the most recent preoperative value, the presence of massive melena, the requirement for endoscopic hemostasis, and/or the need for blood transfusion [15].

T (Timing): Preoperative, intraoperative, and postoperative variables were used to predict the occurrence of delayed bleeding during the post‐ESD follow‐up period.

S (Setting): The predictive model is intended to assess the risk of delayed bleeding following colorectal ESD, thereby facilitating the implementation of targeted preventive interventions.

### 2.3 Study Selection and Screening

Literature screening was undertaken independently by two researchers, with duplicate entries removed via EndNote X9 software. A primary filter was applied through title and abstract review, followed by a secondary screening based on full‐text evaluation to decide on the final inclusion of each study. Exclusion reasons for every article during both screening phases were recorded, and any existing discrepancies were settled by a third researcher.

### 2.4 Data Extraction and Quality Assessment

Following the CHARMS checklist [14], two authors carried out independent data extraction with a standardized extraction table. Relevant information, including study attributes, outcome measures, predictive variables, and model performance, was extracted systematically. All performance evaluation metrics proposed in each study were adopted to capture the predictive efficacy of the constructed models.

Two authors independently evaluated the methodological quality of included studies by employing the Predictive Model Risk of Bias Assessment Tool (PROBAST) [16]. This systematic review focuses on prediction models for delayed bleeding after colorectal ESD. PROBAST comprises four domains, which incorporate 20 signaling questions to assess the risk of bias and applicability of predictive models. These four domains are specified as follows: (1) Participants: the origins of research data and the inclusion/exclusion criteria for enrolled subjects; (2) Predictors: the definition of predictive factors as well as their corresponding measurement approaches, procedures, and time points; (3) Outcome: the operational definition of the target outcome and its relevant measurement methods, implementation processes, and time nodes; (4) Analysis: the appropriateness of statistical handling for key variables involved in model construction. Each signaling question is fact‐based and allows for responses of “yes” (Y), “probably yes” (PY), “no” (N), “probably no” (PN), or “no information” (NI). The overall risk of bias and concerns regarding the clinical applicability of each predictive model were categorized into three levels: low, high, or unclear [16]. Any disagreements arising during the assessment process were resolved through consultation with a third independent researcher.

### 2.5 Data Synthesis

A tabular descriptive analysis was performed to synthesize the core characteristics of the established predictive models. Specifically, the tabulated information encompassed studies, study design, participants, age, main outcome, diagnostic criteria, sample size, predictors included, statistical methods, and predictive performance. For evaluating the performance of these models in forecasting delayed bleeding after colorectal ESD, two key dimensions were adopted: discrimination, quantified by AUC or C‐statistic, and calibration, assessed via calibration curves or the Hosmer‐Lemeshow (H‐L) test. Additionally, certain studies further reported on the models’ sensitivity, specificity, and clinical applicability, which were verified through decision curve analysis. Owing to the pronounced heterogeneity in predictive factors and participant characteristics across the included models, a narrative synthesis and descriptive summary were employed for all outcomes, with no quantitative meta‐analysis conducted throughout the process.

## 3 Result

We retrieved 25,177 records through a systematic search. After removing the duplicate studies, the titles and abstracts of 12,741 articles were read for eligibility screening, of which 53 met the eligibility criteria. Next, upon reading the full text for screening, we excluded 21 studies with outcomes other than delayed bleeding after colorectal ESD, 13 studies that were not modeled, 10 studies that did not provide diagnostic criteria for delayed bleeding, four studies for which data could not be extracted, and five studies that were included in our systematic review [9, 10, 11, 12, 13].

### 3.1 Description of Included Models

#### 3.1.1 Characteristics of Model Derivation

Five models predictive of the risk of delayed bleeding after colorectal ESD were extracted from the included studies. An overview of the development and performance characteristics of the included prediction models is presented in Table 1. The reported incidence of delayed bleeding after colorectal ESD ranged from 2.90% to 9.90%. Four studies were retrospective cohort studies [9, 10, 11, 12], and one was a prospective cohort study [13]. Sample sizes ranged from 302 to 1189. The number of predictors for these models ranged from 3 to 5. Logistic regression analysis was used to establish predictive models for all included studies.

**TABLE 1:** Overview of model development for included prediction models.

Author (year), country	Study design	Participants	Age**^a^**	Main outcome	Diagnostic criteria	Cases/sample size (%)	Predictors in final model	Performance**^b^**
Qian (2025), China	Retrospective cohort	Aged 18 years and older, after colorectal ESD	Age ≥60 years (59.6%)	Delayed postoperative bleeding	Resection site bleeding 12–14 days post‐ESD, with hematochezia and Hb reduction>20 g/L	30/302 (9.90%)	Number of lesions, lesion size, lesion location, degree of submucosal fibrosis, intraoperative bleeding	AUC: 0.831 (95%CI 0.717–0.944), Specificity: 96.13%, Sensitivity: 60.00%, H‐L test: *x* ^2^ = 1.975, *p* = 0.853
Bian (2024), China	Retrospective cohort	Aged 18 years and older, after colorectal ESD	58.40 ± 10.63	Delayed bleeding	Postoperative Hb ≤ 2 g/dL lower than preoperative level or persistent rectal bleeding	37/412 (8.98%)	The location of the lesion in the rectum, a large lesion, prolonged surgery time	C‐index: 0.89 (95%CI 0.87–0.94), DCA: 1%‐70%
Albouys (2023), France	Prospective cohort	Aged 18 years and older, after colorectal ESD	69.00 ± 10.00	Delayed bleeding	Post‐ESD bleeding (hematochezia) ≥6 h to 30 days post‐procedure requiring prolonged hospitalization, readmission, repeat endoscopy/surgery/angiography, or blood transfusion	75/940 (8.00%)	Age ≥75 years, use of antithrombotics, rectal location, size >50 mm, American Society of Anesthesiologists (ASA) score of III or IV	AUC: 0.751 (95%CI 0.690–0.812), Calibration was fair, H‐L test: *x* ^2^ = 7.652, *p* = 0.47, discrimination slope: 0.07 (95%CI 0.04–0.13)
Li (2022), China	Retrospective cohort	Aged 18 years and older, after colorectal ESD	65.9 ± 5.98	Delayed bleeding	Postoperative dizziness, hematemesis, melena, and hemoglobin loss>20 g/L	37/406 (9.10%)	Lesion in the rec⁃ tum, lesion diameter ≥3 cm, severe fibrosis of the submucosal lesion, obvious intraoperative bleeding	C‐index: 0.819
Seo (2019), Korea	Retrospective cohort	Aged 18 years and older, after colorectal ESD	63.0 (IQR 55.0–69.5)	Delayed bleeding	Post‐ESD bleeding requiring emergency department visit, hospitalization, or re‐intervention (repeat colonoscopy ± endoscopic hemostasis, angiography, or surgery)	34/1189 (2.90%)	Tumor location in the rectosigmoid colon, large tumor (30 mm), use of antiplatelet agents except for aspirin alone	AUC: 0.726 (95%CI 0.645–0.808), calibration curve: slope = 0.914, intercept = 0.002, H‐L test: *x* ^2^ = 1.482, *p* = 0.830

ESD = endoscopic submucosal dissection, Hb = hemoglobin, AUC = area under the curve, CI = confidence interval, H‐L test = Hosmer‐Lemeshow test, DCA = decision curve analysis.

^a^ Ages are shown as mean ± SD, median (IQR, range), or percentage according to the reported data.

^b^ C‐index, sensitivity (%), specificity (%), PPV/NPV (%), calibration slope, other (95%CI, if reported). Generally, we consider AUC = 0.5–0.7 as poor discrimination, 0.7–0.8 as moderate discrimination, 0.8–0.9 as good discrimination, and 0.9–1.0 as excellent discrimination.

#### 3.1.2 Included Predictors

The most commonly used predictors were lesion location, lesion size, use of antithrombotics, intraoperative bleeding, and degree of submucosal fibrosis, which appeared in five models, five models, two models, two models, and two models, respectively. Other commonly used predictors included anesthesiologist's score, age≥75 years, prolonged surgery time, and number of lesions, which were used once (Figure 2).

**FIGURE 2:**
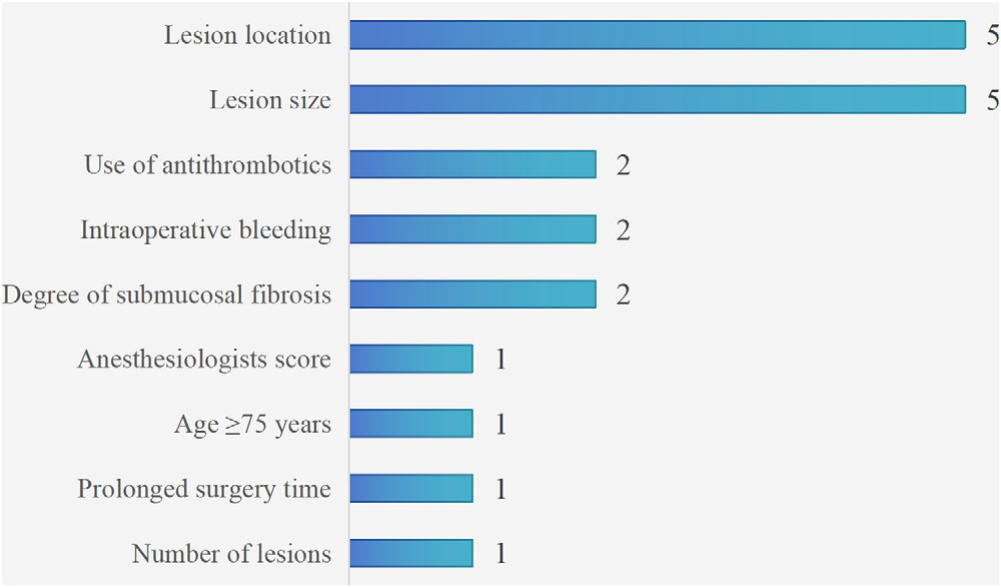
Summary of predictors present in included models.

#### 3.1.3 Model Performance in the Derivation Set

In the derivation set, each of the five models reported AUCs or C‐statistics for the model, with a reporting range of 0.726–0.890. The calibration of five models was reported; three studies were presented as calibration curves, and four studies were presented as H‐L good‐of‐fit tests, all of which showed good performance.

### 3.2 Model Validation

Table 2 shows an overview of model validation for the included prediction models. Four models were only internally validated [9, 10, 11, 13], and one model was only externally validated [12]. The AUCs or C‐statistics for internal validation reports are between 0.739 and 0.870. The reporting range for external validation is 0.634.

**TABLE 2:** Overview of model validation for included prediction models.

Model	Citation of validation (year)	Type of validation	Participants	Age**^a^**	Diagnostic criteria	Cases/sample size (%)	Performance**^b^**
Qian's model (2025)	Qian (2025)	Internal validation	Aged 18 years and older, after colorectal ESD	Age ≥60 years (59.6%)	Resection site bleeding 12–14 days post‐ESD, with hematochezia and Hb reduction>20 g/L	30/302 (9.90%)	AUC: 0.821 (95%CI 0.664–0.979), Specificity: 75.82%, Sensitivity: 90.00%, H‐L test: *x* ^2^ = 8.510, *p* = 0.203
Bian's model (2024)	Bian (2024)	Internal validation	Aged 18 years and older, after colorectal ESD	59.13 ± 10.52	Postoperative Hb ≤ 2 g/dL lower than preoperative level or persistent rectal bleeding	‐/175 (‐%)	AUC: 0.87
Albouys's model (2023)	Albouys (2023)	Internal validation	Aged 18 years and older, after colorectal ESD	69.00 ± 10.00	Post‐ESD bleeding (hematochezia) ≥6 h to 30 days post‐procedure requiring prolonged hospitalization, readmission, repeat endoscopy/surgery/angiography, or blood transfusion	75/940 (8.00%)	AUC: 0.739 (95%CI 0.671–0.805)
Li's model (2022)	Li (2022)	Internal validation	Aged 18 years and older, after colorectal ESD	65.9 ± 5.98	Postoperative dizziness, hematemesis, melena, and hemoglobin loss >20 g/L	37/406 (9.10%)	AUC: 0.839 (95%CI 0.778–0.901), the slope of the calibration curve approached 1 (*x* ^2^ = 7.962, *p* = 0.381)
Seo's model (2019)	Seo (2019)	External validation	Aged 18 years and older, after colorectal ESD	63.0 (IQR 55.0–69.5)	Post‐ESD bleeding requiring emergency department visit, hospitalization, or re‐intervention (repeat colonoscopy ± endoscopic hemostasis, angiography, or surgery)	10/415 (2.41%)	AUC: 0.634 (95%CI 0.472–0.796), H‐L test: *x* ^2^ = 1.666, *p* = 0.435

ESD = endoscopic submucosal dissection, Hb = hemoglobin, AUC = area under the curve, CI = confidence interval, H‐L test = Hosmer‐Lemeshow test, IQR = interquartile range.

^a^ Age is shown as mean ± SD, median (IQR, range), or percentage according to the reported data.

^b^ C‐index, sensitivity (%), specificity (%), PPV/NPV (%), calibration slope, other (95%CI, if reported). Generally, we consider AUC = 0.5–0.7 as poor discrimination, 0.7–0.8 as moderate discrimination, 0.8–0.9 as good discrimination, and 0.9–1.0 as excellent discrimination.

### 3.3 Risk of Bias and Applicability

The overall and domain‐specific results of risk of bias and applicability for the five included studies are presented in Table 3. All studies were rated as having high risk of bias, indicating methodological limitations in the model development or validation processes. For the participants domain, four studies were at high risk of bias, mainly due to the use of retrospective data from restricted or poorly defined source populations and the reliance on existing records without standardized collection protocols, raising concerns about both selection and measurement bias per PROBAST signaling questions [9, 10, 11, 12]. For the predictors domain, all five studies had unclear risk of bias. None of the studies reported whether the investigators assessing predictor variables were blinded to the patients’ delayed bleeding outcomes; no relevant information regarding the use of blinding was identified [9, 10, 11, 12, 13]. For the outcome domain, all five studies were at high risk of bias. Among them, four studies were retrospective in nature, such that investigators could not be blinded to predictor information when ascertaining outcomes and inevitably had access to other patient data (i.e., predictor variables) [9, 10, 11, 12]. One study was a post hoc analysis of a prospectively registered database; although data were collected prospectively, investigators had full access to all predictor and outcome data for all patients when performing statistical analyses and examining associations between delayed bleeding and candidate predictors [13]. For the analysis domain, all five studies were at high risk of bias. One study carried a risk of inadequate sample size [11]. None of the five studies reported the presence or handling of missing data for predictor or outcome variables in the development or validation cohorts [9, 10, 11, 12, 13]. All five studies selected predictors using univariate screening methods [9, 10, 11, 12, 13]. Three studies did not report the presence of data complexity or whether potential data complexity was assessed or accounted for in analyses [10, 11, 13]. Two studies did not employ rigorous internal validation approaches such as cross‐validation [10, 11].

**TABLE 3:** PROBAST results of included studies.

Study Author (year)	Study type	ROB				Applicability			Overall	
		Participants	Predictors	Outcome	Analysis	Participants	Predictors	Outcome	ROB	Applicability
1‐Qian (2025)	B	—	?	—	—	+	+	+	—	+
2‐Bian (2024)	B	—	?	—	—	+	+	+	—	+
3‐Albouys (2023)	B	+	?	—	—	+	+	+	—	+
4‐Li (2022)	B	—	?	—	—	+	+	+	—	+
5‐Seo (2019)	B	—	?	—	—	+	+	+	—	+

PROBAST = Prediction Model Risk of Bias Assessment Tool; ROB = risk of bias.

A indicates“development only”; B indicates “development and validation in the same publication”; C indicates “validation only.”

+ indicates low ROB/low concern regarding applicability; − indicates high ROB/high concern regarding applicability;? indicates unclear.

ROB/unclear concern regarding applicability.

Overall, the five studies carried low concern regarding applicability. The study populations and clinical designs of the included prediction model studies were highly consistent with the research question of this systematic review. Definitions, measurement approaches, and timing of predictor assessments were congruent with the prespecified criteria of this review. Similarly, outcome definitions, follow‐up intervals, and statistical analytical methods were aligned with the prespecified standards set for this systematic review.

## 4 Discussion

ESD has become the minimally invasive gold standard for en bloc resection of large superficial neoplasms [17]. However, delayed bleeding after colorectal ESD remains an unpredictable major adverse event, highlighting an urgent need to establish accurate risk stratification models to guide individualized prophylaxis and postoperative management. This systematic review included five studies, encompassing five prediction models for delayed bleeding after colorectal ESD. Assessment using the PROBAST tool revealed a high risk of bias across all included studies, while concerns regarding applicability were low. These findings indicate that although existing models address clinically relevant questions, they are limited by critical methodological flaws and are not yet suitable for direct translational application.

In the present study, the PROBAST was used to evaluate the methodology of the included studies. The results demonstrated that all five included studies had a high risk of bias, with substantial methodological limitations that may lead to inaccurate and unreliable findings. First, the included studies were predominantly retrospective in design, and none clearly reported blinding to predictor information during outcome assessment. However, because delayed bleeding is a relatively objective outcome, the impact of diagnostic suspicion bias is likely limited. Nonetheless, we recommend that future studies clearly document which variables were known to outcome assessors and, where feasible, implement blinding of outcome adjudicators to the predicted risk scores rather than to individual predictors. Importantly, rather than favoring prospective over retrospective designs per se, future research should prioritize methodological rigor, including clear source population definitions, standardized variable ascertainment, and appropriate handling of missing data. Well‐designed retrospective studies using real‐world data are also widely accepted and valuable in this field. Second, most studies did not adequately report missing data. Several studies arbitrarily excluded patients without complete data, which not only introduced selection bias but also reduced the generalizability of findings. Rather than relying on automatic sample exclusion via statistical software (i.e., complete‐case analysis) during analysis [14], validated missing data methods such as multiple imputation should be applied to minimize bias and improve the robustness of estimates. Third, all included studies selected variables for multivariable regression models based on statistical significance in univariate analysis (*p* < 0.05). Although Albouys et al. [13] used a more liberal threshold of *p* < 0.2, such variable selection strategies relying on univariate P‐values remain limited in two aspects: on the one hand, they may exclude variables that are not statistically significant in univariate analysis but have important predictive potential when combined with other variables; on the other hand, they may include variables showing significant associations by chance, thereby increasing the risk of model overfitting. Finally, many studies did not mention or address the presence of data complexity. We acknowledge, however, that exploring complex structures, such as interactions and subgroup heterogeneity, requires a sufficient number of outcome events to avoid overfitting. We therefore suggest that such explorations be reserved for studies with adequate sample sizes and conducted as pre‑planned sensitivity analyses, preferably using penalized regression techniques to maintain model robustness.

Core metrics for evaluating prediction model performance generally include discrimination and calibration. Discrimination can be reflected by indices such as the C‐statistic, whereas calibration is commonly assessed using the H‐L goodness‐of‐fit test [14]. In the present systematic review, the AUC was the most frequently used index for discrimination when evaluating the performance of the included prediction models. Values closer to 1 indicate superior predictive performance. Among the five prediction models included, the AUC values during the development phase ranged from 0.726 to 0.890, with 3 models demonstrating an AUC greater than 0.8 [9, 10, 11]. These findings suggest that the models exhibited favorable discriminative ability and could relatively accurately predict the probability of delayed bleeding in patients undergoing colorectal ESD. Regarding model calibration, only 3 models presented calibration results using the H‐L goodness‐of‐fit test [9, 12, 13], while the remaining models demonstrated calibration performance via calibration plots without providing quantitative calibration assessment data. Given that the included models generally lacked standardized external validation and reporting of existing performance indices was incomplete, future studies should fully report core performance indices, including discrimination and calibration, and perform rigorous external validation. Only in this way can the clinical translation of high‐quality prediction models be promoted, providing reliable evidence for risk stratification and individualized intervention of delayed bleeding after colorectal ESD.

The predictors included in the models were all derived from routinely recorded clinical data, showing favorable availability, which facilitates subsequent external validation and clinical translation. The most common predictors in the models were lesion size and lesion location, suggesting that these two factors have become core features in current model development. The next most frequent predictors were intraoperative bleeding, use of antithrombotics, and degree of submucosal fibrosis. By contrast, advanced age, prolonged surgery time, number of lesions, and anesthesiologists’ score were only included in a few models, so consistent conclusions could not be drawn. This may be partly limited by the sample size and variable coverage in each study, and it cannot be ruled out that the effects of these factors are relatively weak and easily masked by core predictors.

The mechanisms underlying each core predictor are as follows: lesion size directly influences the area of the postoperative mucosal defect. Studies have demonstrated that each 10‑mm increase in wound diameter is associated with an approximately 30% elevated risk of delayed bleeding [18, 19]. Regarding anatomical location, the rectum and right colon have abundant blood supply and dense lymphatic drainage, resulting in higher postoperative wound exudation and reperfusion pressure. The distal rectum is under continuous compression from the anal sphincter, which may also disturb local revascularization [20, 21]. This may explain why all three models with an AUC > 0.8 included both variables‐they are critical contributors to model discrimination. Intraoperative bleeding serves as a direct indicator reflecting the difficulty of submucosal dissection and the quality of wound management. Repeated oozing or inadequate hemostasis can lead to poor formation of electrocoagulation eschars. During the postoperative inflammatory phase, hyperfibrinolysis and eschar sloughing tend to occur, thereby inducing delayed bleeding [21]. Furthermore, the definition of intraoperative bleeding remains inconsistent across studies: some adopt a quantitative threshold for blood loss, while others rely on the operator's subjective grading. Standardized definitions of this indicator should be established in future research. Antithrombotic agent use is a well‐established risk factor for bleeding events. However, substantial heterogeneity exists among studies in terms of drug categories and discontinuation protocols, leading to inconsistent estimates of its effect across different populations. The degree of submucosal fibrosis is often associated with the depth of lesion invasion or previous biopsy history. Fibrotic areas exhibit reduced tissue compliance, which not only limits the dispersion of submucosal injection fluid and results in poor lesion lifting, thereby increasing dissection difficulty, but also distorts vascular architecture and impairs vessel wall compliance within the fibrotic stroma, markedly compromising the efficacy of electrocoagulation hemostasis [22, 23]. These dual mechanisms jointly increase the risk of secondary bleeding. In summary, the predictive value of core features, including lesion size and lesion location, has been preliminarily confirmed, providing a foundation for subsequent model simplification and optimization. Future studies should retain core variables while actively exploring more timely predictive indicators, such as dynamic parameters of coagulation function and radiomic features of submucosal vessels, to improve model robustness and practicality in real‑world clinical decision‑making. The clinical utility of these models should be interpreted in light of when their predictors become available. In our included studies, models incorporating intraoperative variables were only applicable after the procedure, making them better suited for guiding postoperative surveillance intensity rather than pre‐procedural decision‐making. In contrast, models restricted to preoperative variables could support informed consent and periprocedural planning. Encouragingly, we observed that lesion size and location, the two most frequently included predictors, are both preoperatively accessible, suggesting that a simplified preoperative screening tool may be feasible. However, none of the included studies explicitly defined the intended timing of model application. Future research should clarify the intended use scenario at the design stage and validate models accordingly. An important question is whether these prediction models offer added clinical value beyond conventional risk assessment based on individual factors. On the one hand, these individual factors are already well recognized and may provide intuitive guidance for clinical decision‐making. On the other hand, prediction models have the theoretical advantage of synthesizing multiple predictors into a quantitative, individualized risk estimate, which may capture complex interactions and provide more precise stratification than any single factor alone.

Nevertheless, existing prediction models still carry certain clinical relevance. First, the predictors included in these models can provide candidate variables and theoretical support for the development of new models in future research. Second, the clinical usability of prediction models represents an important dimension for evaluating their translational potential. Some variables, such as the ASA physical status classification, require additional assessments, which not only increases the burden on users but also introduces interobserver variability and measurement bias, thereby impairing model stability and consistency. Third, few studies have evaluated the clinical utility and application benefits of these models. In light of the methodological limitations identified in this review, we emphasize that researchers should fully familiarize themselves with the PROBAST guideline prior to model development. Future studies are encouraged to validate and optimize the current models or develop novel ones under more rigorous methodological standards.

### 4.1 Limitations

This systematic review has several potential limitations. First, significant heterogeneity existed among the included studies in terms of data sources, variable definitions, and statistical methodologies, which precluded a formal meta‐analysis of predictive model performance. Second, only studies published in the English language were included, and conference abstracts and unpublished data were excluded; therefore, the possibility of publication bias and missing relevant literature cannot be ruled out. Third, most of the included prediction models lacked sufficient internal validation and rigorous external validation, especially large‑sample and multicenter validation. The absence of robust external verification may compromise the reliability and generalizability of the current evidence.

## 5 Conclusion

In summary, our systematic review identified five studies describing five predictive models for delayed bleeding in colorectal ESD patients. There are a limited number of models for delayed bleeding in colorectal ESD patients of all ages. The most commonly used predictors were lesion location and lesion size. Discrimination in derivation cohorts ranged from 0.726 to 0.890, internal validation AUCs ranged from 0.739 to 0.870, and the externally validated model achieved an AUC of 0.634. According to PROBAST, all five included studies that developed or validated predictive models were evaluated as having a high risk of bias (with low concerns regarding applicability), and therefore require cautious interpretation.

## Author Contributions

**Conceptualization**: Da Miao. **Data curation**: Zhuoer Hou and Yao Ge. **Formal analysis**: Zhuoer Hou and Yao Ge. **Investigation**: Da Miao. **Supervision**: Haiou Qi. **Writing – original draft**: Zhuoer Hou. **Writing – review & editing**: Da Miao.

## Funding

This work was supported by the Zhejiang Provincial Medical and Health Science Technology Program 2025 (2025KY888).

## Ethics Statement

Not applicable.

## Consent

Not applicable.

## Conflicts of Interest

The authors declare no conflicts of interest.

## Supporting information




**Supporting File 1**: deo270456‐sup‐0001‐SupMat.docx

## Data Availability

All data are in the manuscript and/or supporting information files.

## References

[1] L. Fuccio , C. Hassan , T. Ponchon , et al., “Clinical Outcomes after Endoscopic Submucosal Dissection for Colorectal Neoplasia: A Systematic Review and Meta‐analysis,” Gastrointestinal Endoscopy 86, no. 1 (2017): 74–86.e17. 10.1016/j.gie.2017.02.024 28254526

[2] L. Kang , “Research Progress on Prediction Models for Postoperative Bleeding Risk after Endoscopic Submucosal Dissection of the Stomach,” Journal of Nursing and Rehabilitation 24, no. 08 (2025): 83–87.

[3] K. Boda , S. Oka , S. Tanaka , et al., “Clinical Outcomes of Endoscopic Submucosal Dissection for Colorectal Tumors: A Large Multicenter Retrospective Study from the Hiroshima GI Endoscopy Research Group,” Gastrointestinal Endoscopy 87, no. 3 (2018): 714–722. 10.1016/j.gie.2017.05.051 28623057

[4] L. C. D. S. Costa , J. O. M. Santos , N. T. Miyajima , et al., “Efficacy Analysis of Endoscopic Submucosal Dissection for the Early Gastric Cancer and Precancerous Lesions,” Arquivos De Gastroenterologia 59, no. 3 (2022): 421–427. 10.1590/S0004-2803.20220300075 36102442

[5] J. Sun , X. Xie , Y. Liu , et al., “Complications after Endoscopic Submucosal Dissection for Early Colorectal Cancer (Review),” Oncology Letters 25, no. 6 (2023): 264. 10.3892/ol.2023.13850 PMC10193368 37216158

[6] P. Russo , S. Barbeiro , H. Awadie , et al., “Management of Colorectal Laterally Spreading Tumors: A Systematic Review and Meta‐analysis,” Endoscopy International Open 07, no. 02 (2019): E239–E259. 10.1055/a-0732-487 PMC6353652 30705959

[7] Y. Xu , S. Zhong , W. Liang , et al., “The Risk Factors for Delayed Bleeding after Endoscopic Resection of Colorectal Tumors: A Meta‐analysis,” Expert Review of Gastroenterology & Hepatology 14, no. 11 (2020): 1083–1092. 10.1080/17474124.2020.1801416 32721191

[8] L. Dong , W. Zhu , X. Zhang , et al., “Does Prophylactic Closure Improve Outcomes after Colorectal Endoscopic Submucosal Dissection? A Systematic Review and Meta‐analysis,” Surgical Laparoscopy, Endoscopy & Percutaneous Techniques 34, no. 1 (2024): 94–100. 10.1097/SLE.0000000000001248 38011072

[9] J. Qian , Y. L. Tao , and S. S. Zheng , “Risk Modeling of Delayed Postoperative Bleeding after Endoscopic Submucosal Dissection for Early Colorectal Cancer and Precancerous Lesions,” World Journal of Gastrointestinal Surgery 17, no. 9 (2025): 107326. 10.4240/wjgs.v17.i9.107326 PMC12476782 41024793

[10] F. Bian , K. Li , G. Bian , et al., “Establishing a Nomogram for Predicting the Risk Factors for Delayed Bleeding after Endoscopic Submucosal Dissection for Colorectal Tumors,” International Journal of Colorectal Disease 39, no. 1 (2024): 200. 10.1007/s00384-024-04783-9 PMC11638303 39666009

[11] L. Jiayan , L. Qianyi , L. Zhishang , et al., “Analysis of Delayed Post‐ polypectomy Bleeding after Endoscopic Submucosal Dissection for Early Colorectal Cancer and Predictive Model Construction,” Journal of Colorectal & Anal Surgery 28, no. 04 (2022): 362–365.

[12] M. Seo , E. M. Song , J. W. Cho , et al., “A Risk‐scoring Model for the Prediction of Delayed Bleeding after Colorectal Endoscopic Submucosal Dissection,” Gastrointestinal Endoscopy 89, no. 5 (2019): 990–998, e2. 10.1016/j.gie.2018.11.029 30521794

[13] J. Albouys , S. Montori Pina , S. Boukechiche , et al., “Risk of Delayed Bleeding after Colorectal Endoscopic Submucosal Dissection: The Limoges Bleeding Score,” Endoscopy 56, no. 02 (2024): 110–118. 10.1055/a-2189-0807 37816392

[14] K. G. M. Moons , J. A. H. De Groot , W. Bouwmeester , et al., “Critical Appraisal and Data Extraction for Systematic Reviews of Prediction Modelling Studies: The CHARMS Checklist,” PLoS Medicine 11, no. 10 (2014): e1001744. 10.1371/journal.pmed.1001744 PMC4196729 25314315

[15] F. F. Bahin , K. N. Rasouli , K. Byth , et al., “Prediction of Clinically Significant Bleeding Following Wide‐Field Endoscopic Resection of Large Sessile and Laterally Spreading Colorectal Lesions: A Clinical Risk Score,” American Journal of Gastroenterology 111, no. 8 (2016): 1115–1122. 10.1038/ajg.2016.235 27296942

[16] K. G. M. Moons , R. F. Wolff , R. D. Riley , et al., “PROBAST: A Tool to Assess Risk of Bias and Applicability of Prediction Model Studies: Explanation and Elaboration,” Annals of Internal Medicine 170, no. 1 (2019): W1. 10.7326/M18-1377 30596876

[17] M. S. Hossain , H. Karuniawati , A. A. Jairoun , et al., “Colorectal Cancer: A Review of Carcinogenesis, Global Epidemiology, Current Challenges, Risk Factors, Preventive and Treatment Strategies,” Cancers 14, no. 7 (2022): 1732. 10.3390/cancers14071732 PMC8996939 35406504

[18] Z. Wu , L. Yuan , S. Zhang , et al., “Risk Prediction for Delayed Bleeding after Endoscopic Submucosal Dissection in Colorectal Precancerous Lesions Using Artificial Intelligence: Retrospective Clinical Trial,” Diseases of the Colon and Rectum 69, no. 4 (2026): 651–661. 10.1097/DCR.0000000000004095 PMC13003935 41441208

[19] J. Arimoto , K. Ohata , J. Tachikawa , et al., “The Management of Direct Oral Anticoagulants for Colorectal Endoscopic Submucosal Dissection: A Multicenter Observational Study,” Digestive Diseases and Sciences 69, no. 3 (2024): 933–939. 10.1007/s10620-024-08316-w 38340260

[20] N. Ogasawara , T. Yoshimine , H. Noda , et al., “Clinical Risk Factors for Delayed Bleeding after Endoscopic Submucosal Dissection for Colorectal Tumors in Japanese Patients,” European Journal of Gastroenterology & Hepatology 28, no. 12 (2016): 1407–1414. 10.1097/MEG.0000000000000723 27512926

[21] E. Albéniz , S. Montori , M. Enguita‐Germán , et al., “External Validation and Optimization of the Limoges Score to Improve the Delayed Bleeding Risk Prediction After Colorectal Endoscopic Submucosal Dissection: The Delayed Bleeding ESD (DEBE) Score,” Endoscopy 58 (2026): 741–752. 10.1055/a-2814-4950 PMC13286675 41871608

[22] N. G. Burgess , A. J. Metz , S. J. Williams , et al., “Risk Factors for Intraprocedural and Clinically Significant Delayed Bleeding after Wide‐field Endoscopic Mucosal Resection of Large Colonic Lesions,” Clinical and Gastroenterological Hepatology 12, no. 4 (2014): 651–661, e3. 10.1016/j.cgh.2013.09.049 24090728

[23] F. Gu , W. Jiang , J. Zhu , et al., “Risk Factors for Unsuccessful Colorectal Endoscopic Submucosal Dissection: A Systematic Review and Meta‐analysis,” Digestive and Liver Disease 56, no. 8 (2024): 1288–1297. 10.1016/j.dld.2023.11.030 38071178

