# Supplementary material for: Prediction Models for Delayed Bleeding After Colorectal Endoscopic Submucosal Dissection: A Systematic Review and Critical Appraisal
Source: DEN Open. 2026 Oct 19;7(1):e70456. doi: 10.1002/deo2.70456 (PMC13678419; doi:10.1002/deo2.70456)
Supplement: Supplementary file 1 [file DEO2-7-e70456-s001.docx]

**Supplemental Material**

**Table S1. Search strategies in each database---2026.3.20**

| **Database** | **Search strategies** | **Results** |
| --- | --- | --- |
| **PubMed** | #1 delayed bleeding[Title/Abstract] OR bleeding[Title/Abstract] OR postoperative bleeding[Title/Abstract] OR delayed hemorrhage[Title/Abstract] OR postoperative hemorrhage[Title/Abstract]---276,546  #2 prediction model[Title/Abstract] OR prediction[Title/Abstract] OR predict model[Title/Abstract] OR risk prediction[Title/Abstract] OR risk factors[Title/Abstract] OR risk assessment[Title/Abstract] OR prognostic model[Title/Abstract] OR model[Title/Abstract] OR nomogram[Title/Abstract]---4,150,578  #3 endoscopy[Mesh]---434,218  #4 endoscopy[Title/Abstract] OR endoscopic[Title/Abstract] OR endoscopic resection[Title/Abstract] OR endoscopic submucosal dissection[Title/Abstract] OR ESD[Title/Abstract] OR endoscopic mucosal resection[Title/Abstract] OR EMR[Title/Abstract]---278,900  #5 #3 OR #4---577,609  #6 #1 AND #2 AND #5---3,725 | 3,725 |
| **Web of Science** | #1 TI=(delayed bleeding OR bleeding OR postoperative bleeding OR delayed hemorrhage OR postoperative hemorrhage) OR AB=(delayed bleeding OR bleeding OR postoperative bleeding OR delayed hemorrhage OR postoperative hemorrhage)---541,841  #2 TI=(prediction model OR prediction OR predict model OR risk prediction OR risk factors OR risk assessment OR prognostic model OR model OR nomogram) OR AB=(prediction model OR prediction OR predict model OR risk prediction OR risk factors OR risk assessment OR prognostic model OR model OR nomogram)---36,235,061  #3 ((TS=(endoscopy)) OR TI=(endoscopy OR endoscopic OR endoscopic resection OR endoscopic submucosal dissection OR ESD OR endoscopic mucosal resection OR EMR)) OR AB=(endoscopy OR endoscopic OR endoscopic resection OR endoscopic submucosal dissection OR ESD OR endoscopic mucosal resection OR EMR)---494,567  #3 #1 AND #2 AND #3---9,159 | 9,159 |
| **Embase** | #1 'delayed bleeding':ti,ab,kw OR bleeding:ti,ab,kw OR 'postoperative bleeding':ti,ab,kw OR 'delayed hemorrhage':ti,ab,kw OR 'postoperative hemorrhage':ti,ab,kw---472,650  #2 'prediction model':ti,ab,kw OR prediction:ti,ab,kw OR 'predict model':ti,ab,kw OR 'risk prediction':ti,ab,kw OR 'risk factors':ti,ab,kw OR 'risk assessment':ti,ab,kw OR 'prognostic model':ti,ab,kw OR 'model':ti,ab,kw OR 'nomogram':ti,ab,kw---5,436,218  #3 'endoscopy'/exp OR endoscopy:ti,ab,kw OR endoscopic:ti,ab,kw OR 'endoscopic resection':ti,ab,kw OR 'endoscopic submucosal dissection':ti,ab,kw OR ESD:ti,ab,kw OR 'endoscopic mucosal resection':ti,ab,kw OR EMR:ti,ab,kw---1,211,705  #4 #1 AND #2 AND #3---9,789 | 9,789 |
| **Cochrane library** | #1 (delayed bleeding OR bleeding OR postoperative bleeding OR delayed hemorrhage OR postoperative hemorrhage):ti,ab,kw (Word variations have been searched)---69,874  #2 (prediction model OR prediction OR predict model OR risk prediction OR risk factors OR risk assessment OR prognostic model OR model OR nomogram):ti,ab,kw (Word variations have been searched)---286,722  #3 MeSH descriptor: [endoscopy] explode all trees---23,108  #4 (endoscopy OR endoscopic OR endoscopic resection OR endoscopic submucosal dissection OR ESD OR endoscopic mucosal resection OR EMR):ti,ab,kw (Word variations have been searched)---38,012  #5 #3 OR #4---41,916  #6 #1 AND #2 AND #5---1,493 | 1,493 |
| **CNKI** | #1 (TKA=delayed bleeding OR bleeding OR postoperative bleeding OR delayed hemorrhage OR postoperative hemorrhage)---412,800  #2 (TKA=prediction model OR prediction OR predict model OR risk prediction OR risk factors OR risk assessment OR prognostic model OR model OR nomogram)---4,252,700  #3 (SU=endoscopy) OR (TKA=endoscopy OR endoscopic OR endoscopic resection OR endoscopic submucosal dissection OR ESD OR endoscopic mucosal resection OR EMR)---108,500  #4 #1 AND #2 AND #3---1,011 | 1,011 |
